# Thyroid Cancer Incidences From Selected South America Population-Based Cancer Registries: An Age-Period-Cohort Study

**DOI:** 10.1200/JGO.17.00024

**Published:** 2017-08-21

**Authors:** Anne Karin da Mota Borges, Adalberto Miranda-Filho, Sérgio Koifman, Rosalina Jorge Koifman

**Affiliations:** **Anne Karin da Mota Borges**, **Sérgio Koifman**,† and **Rosalina Jorge Koifman**, National Public Health School, Oswaldo Cruz Foundation; **Anne Karin da Mota Borges**, Brazilian National Cancer Institute, Rio de Janeiro, Brazil; and **Adalberto Miranda-Filho**, International Agency for Research on Cancer, Lyon, France.

## Abstract

**Purpose:**

The incidence of thyroid cancer (TC) has increased substantially worldwide. However, there is a lack of knowledge about age-period-cohort (APC) effects on incidence rates in South American countries. This study describes the TC incidence trends and analyzes APC effects in Cali, Colombia; Costa Rica; Goiânia, Brazil; and Quito, Ecuador.

**Materials and Methods:**

Data were obtained from the Cancer Incidence in Five Continents series, and the crude and age-standardized incidence rates were calculated. Trends were assessed using the estimated annual percentage change, and APC models were estimated using Poisson regression for individuals between age 20 and 79 years.

**Results:**

An increasing trend in age-standardized incidence rates was observed among women from Goiânia (9.2%), Costa Rica (5.7%), Quito (4.0%), and Cali (3.4%), and in men from Goiânia (10.0%) and Costa Rica (3.4%). The APC modeling showed that there was a period effect in all regions and for both sexes. Increasing rate ratios were observed among women over the periods. The best fit model was the APC model in women from all regions and in men from Quito, whereas the age-cohort model showed a better fit in men from Cali and Costa Rica, and the age-drift model showed a better fit among men from Goiânia.

**Conclusion:**

These findings suggest that overdiagnosis is a possible explanation for the observed increasing pattern of TC incidence. However, some environmental exposures may also have contributed to the observed increase.

## INTRODUCTION

Thyroid cancer (TC) incidence varies greatly worldwide with major differences in age-standardized incidence rates (ASRs) according to region and sex.^1,2^ A substantial increase in TC incidence in various high-income countries over the past 30 years has been reported in the literature.^3-8^ Several possible explanations have been put forward to account for this increase.^6^ However, controversy still exists as to the contribution of risk factors to this temporal trend, mainly because, with the exception of exposure to ionizing radiation during childhood and among young women, their role in the development of TC has still not been fully elucidated.^9^ Given that the increasing trend in TC incidence and the increased use and improvement in the sensitivity of diagnostic technologies are coincident, the hypothesis of overdiagnosis has been proposed to explain the observed increase.^6,8,10-12^ In the past, malignant thyroid nodules were diagnosed in patients who presented with a visible mass or compressive symptoms. In the late 1980s, the advent of ultrasonography and ultrasound-guided fine-needle biopsy enabled the detection of millimeter-sized nodules.^8^

Studies examining the effects of age, period, and cohort on TC incidence have yielded somewhat divergent findings.^5,12-15^ Although some authors have suggested that increased incidence of TC may be associated with higher diagnostic intensity,^5,12^ others point out that a variety of environmental factors may contribute to this increasing trend.^13-15^ These studies were carried out in high-income populations and thus reflect a reality different from that of middle- and low-income populations, given that the diagnosis of TC is highly dependent on technology and, therefore, on the access to health care services. In view of the lack of research into age-period-cohort (APC) effects on TC incidence in South America, the current study aims to assess the temporal patterns of TC incidence and estimate the effects of age, period, and birth cohort in four regions covered by population-based cancer registries (PBCRs) in the periods from 1983 to 2007 (Cali in Colombia and Costa Rica) and 1988 to 2007 (Goiânia in Brazil and Quito in Ecuador).

## MATERIAL AND METHODS

### Data Sources

Data on the number of patients with TC in each region, the respective year and age of diagnosis, and the size of at-risk populations were obtained from the Cancer Incidence in Five Continents series, volumes VI to X, published by the International Agency for Research on Cancer.^1,16-19^ Cancer Incidence in Five Continents data are obtained from high-quality cancer registries of a particular country or region (Appendix Table A1). The four PBCRs were chosen because they were evaluated by a rigorous editorial process and reached the highest level of quality. Furthermore, they are the only registries in South America with at least 20 years of uninterrupted time series.

### Statistical Analysis

For each region, crude incidence rates and ASRs were calculated, expressed per 100,000 person-years at risk and stratified by sex for each year and for each 5-year study period. ASRs were calculated using the direct method and world standard population.^20,21^

Temporal trends in ASRs were assessed using the estimated annual percentage change (EAPC), which was calculated using the following formula: EAPC = 100[(*e^m^*) − 1], where *m* was estimated using a regression model with the logarithm of the ASR as the dependent variable and calendar year as the explanatory variable. The selection of the best model was based on the result of a permutation test.^22^ EAPC was considered statistically significant at the *P* < .05. The models were estimated using the Joinpoint Regression Program, version 4.4.0.0.^23^ The temporal trend curves were performed using Stata statistical software (Stata, College Station, TX) and were smoothed using locally weighted scatterplot smoothing with a smoothing coefficient of 0.5.

To separate the effects of age, period, and birth cohort on TC incidence rates, APC models were estimated. To this end, age was grouped into 5-year intervals starting at 20 to 24 years and ending with 75 to 79 years. The study periods were also grouped into 5-year intervals as follows: four periods for Goiânia and Quito (1988 to 1992, 1993 to 1997, 1998 to 2002, and 2003 to 2007) and five periods for Cali and Costa Rica (1983 to 1987,1988 to 1992, 1993 to 1997, 1998 to 2002, and 2003 to 2007). Birth cohorts were estimated by subtracting the midpoint of the 5-year age group from the corresponding 5-year period.

APC effects with their respective rate ratios (RRs) and 95% CIs were calculated using the Poisson regression technique. In this Poisson model, the APC effects act multiplicatively on the rate. Thus, the logarithm of the expected rate is a linear function of the effects of age, period, and cohort,^24-27^ given as:

$$\ln\left(E\left[r_{ij}\right]\right)=\text{ln}\left(\frac{\theta_{ij}}{N_{ij}}\right)=\mu +\alpha_{i}+\beta_{j}+\gamma_{k}$$

Where (*E*[*r_ij_*]) denotes the expected incidence rate in age group *i* and period *j*,$\theta_{ij}$ the number of cases in age *i* and period *j*, and $N_{ij}$ the population at risk in age *i* and period *j*; *μ* is the average value of effects (intercept); $\alpha_{i}$ is the effect of age group *i*, $\beta_{j}$ is the effect of time period *j*, and $\gamma_{k}$ is the effect of cohort *k*.^24,26^

The main problem when estimating APC effect parameters is the exact linear dependency among the factors of age, period, and cohort. This dependency impedes the estimation of the three effects using a full model, which is called nonidentifiability. Various solutions have been proposed to overcome this problem. In the current study, the parameterization method developed by Holford^27^ was chosen, which estimates APC effect parameters using the following estimable functions: deviations, curvatures, and drift. This method was applied to allow us to interpret the period and the cohort effects as a RR relative to the reference period (ie, 1983 to 1987 for Cali and Costa Rica and 1988 to 1992 for Goiânia and Quito). The inclusion of the drift with the period effect makes the age effect interpretable as the age-specific rates in the reference period adjusted by the cohort effect. The cohort effect function was set at 0 on average with 0 slope, which is interpretable as the cohort-related RR, after adjustment for age and period.

Natural cubic spline function was used to fit the APC model. The optimal number of knots was selected by adding an increasing number of knots at subsequent quantiles of age, period, and cohort, respectively. Goodness of model fit was tested using the deviance, which was defined as two times the log-likelihood ratio of the estimated model compared with the full model. The contribution of the effects was tested by comparing the deviance of the specific effect model with the full model (APC). The findings were considered statistically significant at *P* < .05. The APC analyses were performed using the statistical software R version 3.3.2, Package Epi 2.0.^28^

## RESULTS

### Geographic and Temporal Patterns

A total of 7,889 patients with TC were registered during the study period among adults between 20 and 79 years old, of whom 84.9% were women and 15.1% were men. ASRs were higher in the last period compared with the first period; ASRs were 3.3 times higher both in women (14.6 *v* 4.4, respectively) and men (3.0 *v* 0.9, respectively) in Goiânia, 3.4 times higher among women (12.4 *v* 3.7, respectively) and 2.2 times higher among men (2.0 *v* 0.9, respectively) in Costa Rica, 2.5 times higher among women (17.0 *v* 6.9, respectively) and 1.5 times among men (3.7 *v* 2.4, respectively) in Quito, and 1.7 times higher among women (11.3 *v* 6.5, respectively) and 1.2 times among men (2.3 *v* 1.9, respectively) in Cali (Table 1).

**Table 1:**
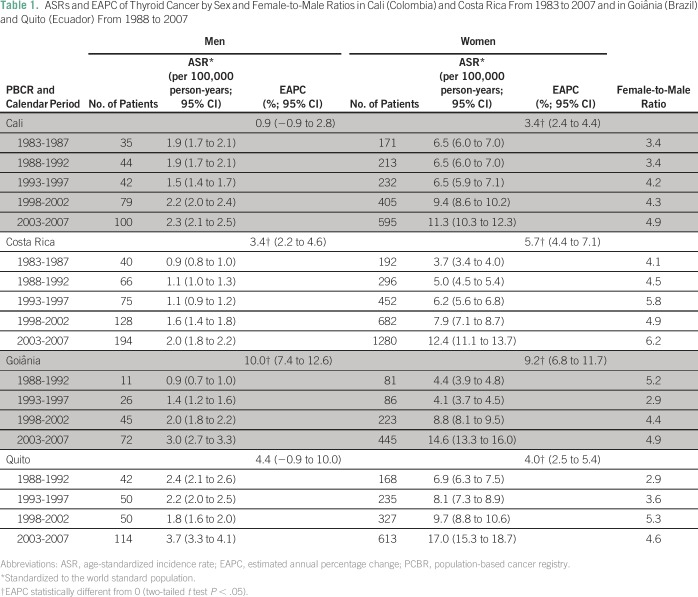
ASRs and EAPC of Thyroid Cancer by Sex and Female-to-Male Ratios in Cali (Colombia) and Costa Rica From 1983 to 2007 and in Goiânia (Brazil) and Quito (Ecuador) From 1988 to 2007

Women showed the highest ASR throughout all periods and in all four regions. The female-to-male ratio reached the highest value in Costa Rica (ie, 6.2) for the period from 2003 to 2007, whereas it was practically homogenous in Goiânia, Quito, and Cali (4.9, 4.6, and 4.9, respectively; Table 1).

**Fig 1:**
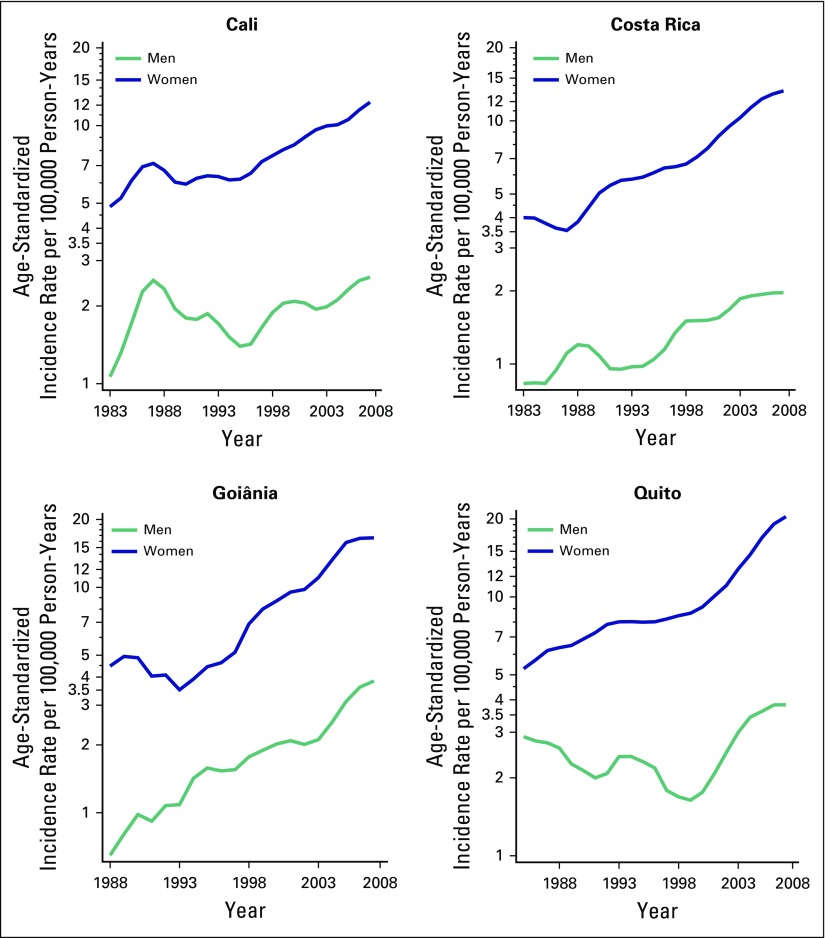
Temporal trends in age-standardized thyroid cancer incidence rates (world standard population) per 100,000 person-years by sex in Cali (Colombia) and Costa Rica from 1983 to 2007 and in Goiânia (Brazil) and Quito (Ecuador) from 1988 to 2007.

Figure 1 shows TC incidence trends according to sex in the four regions. An increase in ASR was observed for both sexes in Goiânia and Costa Rica, whereas in Quito and Cali the increase was only statistically significant among women. In Goiânia, EAPC was slightly higher among men than women (10% *v* 9.2%, respectively), whereas in Costa Rica, the EAPC was substantially higher among women than men (5.7% *v* 3.4%, respectively; Table 1).

### Effects of Age, Period, and Birth Cohort

**Fig 2:**
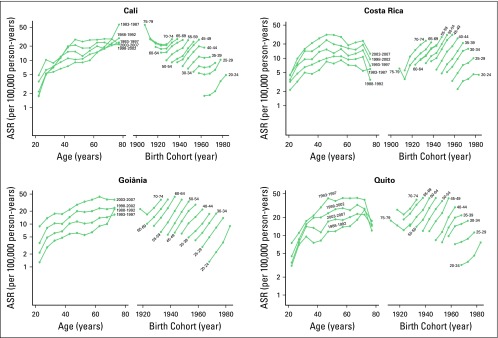
Age-specific incidence rates (ASRs) of thyroid cancer by period of diagnosis and birth cohort among women in Cali (Colombia) and Costa Rica from 1983 to 2007 and in Goiânia (Brazil) and Quito (Ecuador) from 1988 to 2007. Rates are expressed in logarithmic scale.

**Fig 3:**
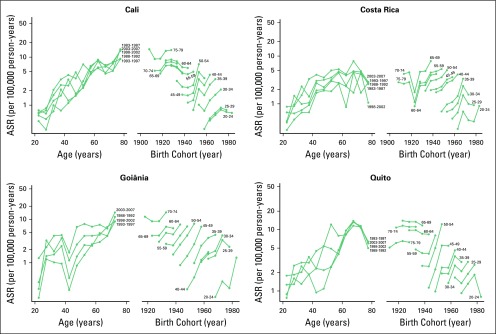
Age-specific incidence rates (ASRs) of thyroid cancer by period of diagnosis and birth cohort among men in Cali (Colombia) and Costa Rica from 1983 to 2007 and in Goiânia (Brazil) and Quito (Ecuador) from 1988 to 2007. Rates are expressed in logarithmic scale.

Figures 2 and 3 show the age- and birth cohort–specific TC incidence rates. Age-specific incidence rates showed a peak shift in age between the first and the last periods of diagnosis, as follows: from 70 to 74 to 60 to 64 years among women and from 70 to 74 to 75 to 79 years among men in Goiânia; from 65 to 69 to 45 to 49 years among women and from 70 to 74 to 65 to 69 years among men in Costa Rica; from 70 to 74 to 55 to 59 years among women and from 60 to 65 to 70 to 75 among men in Quito; and from 75 to 79 to 70 to 74 years among women in Cali. The peak of the age-specific incidence rate in men from Cali occurred in age group 75 to 79 years in both periods. Birth cohort–specific incidence rates consistently increased among women in all four regions and all age groups, except for the age group of 75 to 79 years in Cali and Quito.

**Fig 4:**
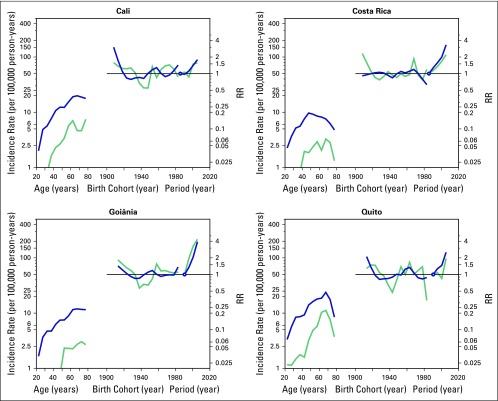
Age, period, and cohort effects on the incidence of thyroid cancer among women (blue) and men (blue-green) in Cali (Colombia) and Costa Rica from 1983 to 2007 and in Goiânia (Brazil) and Quito (Ecuador) from 1988 to 2007. RR, rate ratio.

Figure 4 shows the relative contributions of age, period, and birth cohort to TC incidence rates. The age effect adjusted by period and cohort effects was statistically significant among women in all age groups and all regions. Incidence rates increased at an earlier age in women than men in all regions, except for Quito. The slope of the curve for women peaks at age 45 to 49 years in Costa Rica and at age 65 to 69 years in Cali, Goiânia, and Quito, whereas it peaks at age 65 to 69 years among men in Costa Rica and Quito and at age 70 to 74 years and 75 to 79 years among men in Goiânia and Cali, respectively. The cohort effect only increased the RRs among women in the 1906 to 1910 and the 1911 to 1915 birth cohorts in Cali, in the 1966 to 1970 birth cohort in Costa Rica, and in the 1911 to 1915, 1916 to 1920, 1956 to 1960, and 1961 to 1965 birth cohorts in Quito. Regarding the period effect, a steady increase was noted in RR across periods among women in all four regions. In comparison with the first period, the RRs in 2003 to 2007 were 3.9, 3.3, 2.5, and 1.8 in Goiânia, Costa Rica, Quito, and Cali, respectively. Increasing RR across periods was also observed among men in Goiânia and Costa Rica. In comparison with the first period, the RRs among men in 2003 to 2007 were 4.3, 2.2, 1.9, and 1.6 in Goiânia, Costa Rica, Quito, and Cali, respectively.

The estimated annual changes (net drift) based on the period and cohort effects for women and men were 3.1% (95% CI, 2.3% to 3.9%) and 2.5% (95% CI, 0.7% to 4.3%) in Cali, 5.9% (95% CI, 5.1% to 6.6%) and 4.0% (95% CI, 2.5% to 5.6%) in Costa Rica, 9.5% (95% CI, 7.7% to 11.3%) and 10.3% (95% CI, 5.9% to 15.0%) in Goiânia, and 6.0% (95% CI, 4.8% to 7.2%) and 3.6% (95% CI, 1.0% to 6.2%) in Quito, respectively.

### Model Evaluation

The APC model was fitted for men and women separately. The results are listed in Table 2. The full model (APC) yielded a better fit than the two-factor models (age-period and age-cohort models) for women in all regions and for men in Quito. However, the age-cohort model showed a better fit for men in Cali and Costa Rica, whereas the age-drift model yielded a better fit for men in Goiânia.

**Table 2:**
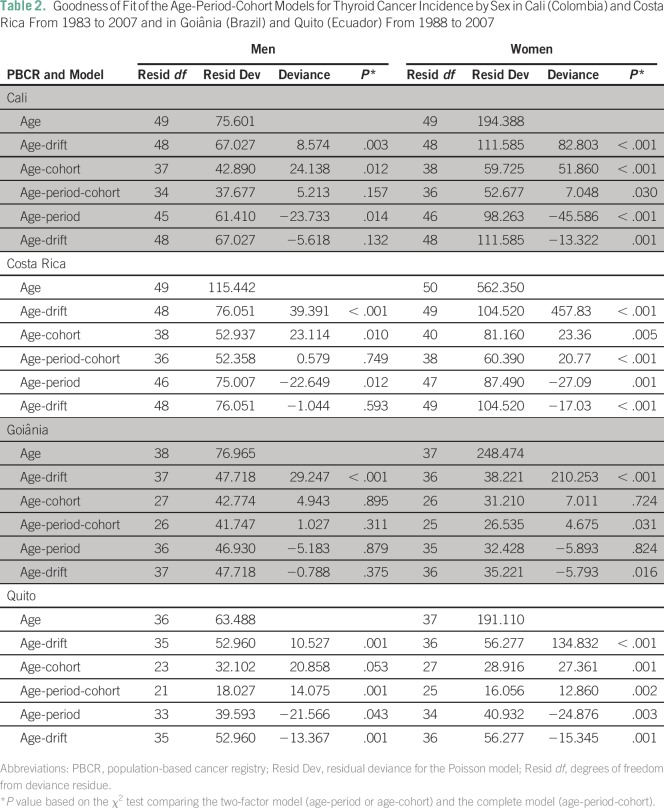
Goodness of Fit of the Age-Period-Cohort Models for Thyroid Cancer Incidence by Sex in Cali (Colombia) and Costa Rica From 1983 to 2007 and in Goiânia (Brazil) and Quito (Ecuador) From 1988 to 2007

## DISCUSSION

The findings of this study showed a substantial increase in TC incidence for both sexes in Cali, Costa Rica, Goiânia, and Quito. Although TC was more common in women than men, incidence increase in Goiânia was slightly more pronounced among men, which is in accordance with a study carried out in the United States in Los Angeles, California.^29^ In contrast, the increase in Costa Rica was greater among women, which is in line with the findings of studies conducted in Australia^3^ and Canada.^5^

Results also revealed a decrease in the age of peak incidence between the first and the last study period among women in all four regions studied. However, except for Costa Rica, the peak age of incidence was higher than that reported by studies using PBCR data for high-income countries.^5,12,13^ A possible explanation for age groups with higher incidence rates being older in low- and middle-income countries may be the later diagnosis in these regions as a result of the difficulties in access to health care services in comparison with high-income countries. In this regard, a study carried out in Wisconsin found a moderate positive correlation between indicators of both socioeconomic status and access to health care services and TC incidence over the period from 1980 to 2004.^30^

The APC model on TC incidence revealed sex similarities and differences. A clear period effect was observed for women and men in all regions. However, the age effect was quite different between sexes, with a peak TC incidence occurring in younger age groups among women in all regions except Quito, where the highest incidence was seen in the same age group for both sexes. Similar findings were reported by Dal Maso et al^12^ regarding the incidence of papillary TC in Italy.

Sex differences in age effect are probably a result of the fact that young and middle-aged women use more health care services than men in the same age group, often as a result of reproductive events and menopause. This may result in sex differences in intensity of screening, thus leading to more timely diagnosis in women.^6,12^ The age effect among men may reflect the fact that men tend to be more concerned about their health at older ages than women.

The observed period effect points to a role of overdiagnosis given the increasing medical surveillance of thyroid nodules and symptoms related to thyroid dysfunction in the past decades.^3,10^ Indeed, the use of ultrasonography and thyroid hormone assays has increased since the 1980s.^31^ A study carried out by Vaccarella et al,^8^ comparing the ASRs between 2003 to 2007 and 1988 to 1992, showed that advances in diagnostic technologies and the increase in medical surveillance of the thyroid gland accounted for 60% of TCs diagnosed in women younger than age 80 years in France, Italy, the United States, and Australia; 30% of TCs diagnosed in Japan; and approximately 50% of TCs diagnosed in the other countries. The percentage of TC diagnoses attributable to changing diagnostic practices was similar for both sexes, although among men, the increase in incidence was slightly smaller and occurred later in time than women.^8^ Overdiagnosis has important medical and socioeconomic implications, given the cost of unnecessary treatment that will provide little benefit and lead to a permanent morbidity associated with thyroidectomy and thyroid hormone replacement. Therefore, these implications should be carefully evaluated, especially among young women.^6,12^

Increased detection afforded by changing diagnostic practices may not be the only explanation for the increase in TC incidence, especially among men in Cali, Costa Rica, and Goiânia. Additional factors that vary with time may have also contributed, at least partially, to the increased TC incidence. For example, the cesium-137 radiologic accident in Goiânia in 1985 may have impacted the number of patients diagnosed with TC because the medical surveillance may have increased in subsequent years.^32,33^ Exposure to ionizing radiation during childhood is the most important risk factor for TC. Therefore, the question that arises is whether there has been an increase in exposure to ionizing radiation among the general public in South America in recent decades. Although there is a lack of population-based research into exposure to ionizing radiation in South America, studies in the United States have reported a significant increase in the per-capita use of x-rays in medical procedures since the 1960s.^34-36^ Mettler et al^35^ found that the per-capita dose of radiation, mostly from medical diagnostic procedures, in the United States has doubled in recent decades. In addition, Fazel et al^36^ suggest that the current pattern of imaging examinations in the United States is exposing individuals to substantial doses of ionizing radiation. Furthermore, epidemiologic studies have shown associations between exposure to diagnostic x-ray examinations and increased risk of TC.^37-40^

TC is one of the few cancers that predominantly affect women. Our findings showed that female-to-male ratios were fairly consistent in all regions, suggesting that female sex hormones may play an important role in TC pathogenesis.^41-43^ This hypothesis suggests the potential importance of endogenous hormones and endocrine disruptors in the development of TC,^44^ as supported by recent studies suggesting that polyhalogenated aromatic hydrocarbons, particularly polybrominated diphenyl ethers, may be associated with the risk of TC because they disrupt thyroid hormones.^44^ In this regard, there has been an increase in human exposure to polybrominated diphenyl ethers and other polyhalogenated aromatic hydrocarbons over recent decades,^45^ which may also have contributed to the observed increase in TC incidence. However, the role of these exposures in thyroid carcinogenesis remains unclear.

Limitations of data from PBCRs may have affected study findings, although the selected PBCRs have been shown to achieve standards of high quality^1,16-19^ and our results are consistent with other population-based studies.^5,12^

It is noteworthy to mention that the findings of this study are not representative of South America as a whole, because only Cali, Costa Rica, Goiânia, and Quito were considered. In addition, the only PBCR that covers the whole country is that of Costa Rica. Cali’s PBCR covers the urban area of the city; Quito’s registry covers the capital city of Ecuador; and Goiânia’s PBCR covers the capital city of the State of Goiás in Brazil. However, it should be highlighted that the current study is the first, to our knowledge, to use APC modeling to examine trends in TC incidence using data from different regions of South America.

Overall, PBCR data showed an increasing trend in TC incidence for both sexes over the past 25 years in Cali and Costa Rica and in the past 20 years in Goiânia and Quito. This increase is largely a result of the period effect, suggesting that overdiagnosis might be one possible explanation for this trend. However, environmental exposures may also have partially contributed to the observed increase, particularly among men in Cali, Costa Rica, and Goiânia.

## Figures and Tables

**Table A1:**
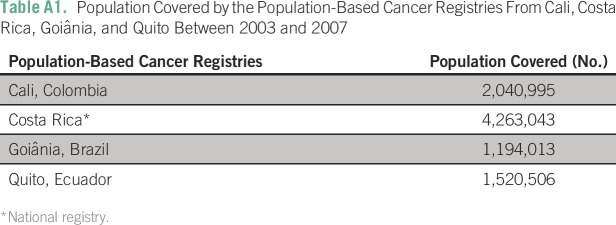
Population Covered by the Population-Based Cancer Registries From Cali, Costa Rica, Goiânia, and Quito Between 2003 and 2007
